# Transcriptomic profiling of taproot growth and sucrose accumulation in sugar beet (*Beta vulgaris* L.) at different developmental stages

**DOI:** 10.1371/journal.pone.0175454

**Published:** 2017-04-13

**Authors:** Yong-Feng Zhang, Guo-Long Li, Xue-Feng Wang, Ya-Qing Sun, Shao-Ying Zhang

**Affiliations:** Sugar Beet Physiological Research Institute, Inner Mongolia Agricultural University, Hohhot, China; Universidade de Lisboa Instituto Superior de Agronomia, PORTUGAL

## Abstract

In sugar beet (*Beta vulgaris* L.), taproot weight and sucrose content are the important determinants of yield and quality. However, high yield and low sucrose content are two tightly bound agronomic traits. The advances in next-generation sequencing technology and the publication of sugar beet genome have provided a method for the study of molecular mechanism underlying the regulation of these two agronomic traits. In this work, we performed comparative transcriptomic analyses in the high taproot yield cultivar SD13829 and the high sucrose content cultivar BS02 at five developmental stages. More than 50,000,000 pair-end clean reads for each library were generated. When taproot turned into the rapid growth stage at the growth stage of 82 days after emergence (DAE), eighteen enriched gene ontology (GO) terms, including cell wall, cytoskeleton, and enzyme linked receptor protein signaling pathway, occurred in both cultivars. Differentially expressed genes (DEGs) of paired comparison in both cultivars were enriched in the cell wall GO term. For pathway enrichment analyses of DEGs that were respectively generated at 82 DAE compared to 59 DAE (the earlier developmental stage before taproot turning into the rapid growth stage), plant hormone signal transduction pathway was enriched. At 82 DAE, the rapid enlarging stage of taproot, several transcription factor family members were up-regulated in both cultivars. An antagonistic expression of brassinosteroid- and auxin-related genes was also detected. In SD13829, the growth strategy was relatively focused on cell enlargement promoted by brassinosteroid signaling, whereas in BS02, it was relatively focused on secondarily cambial cell division regulated by cytokinin, auxin and brassinosteroid signaling. Taken together, our data demonstrate that the weight and sucrose content of taproot rely on its growth strategy, which is controlled by brassinosteroid, auxin, cytokinin, and gibberellin.

## Introduction

As one of the most important sugar crops, sugar beet (*Beta vulgaris ssp*. *vulgaris*) provides up to 30% of the world’s sugar production. Traditional breeding on sugar beet has been aimed to increase the sucrose content and yield of taproot, as well as the resistance to biotic or abiotic stress. Since the start of sugar beet breeding 200 years ago, an increase of 10% in sugar content, as well as a great improvement in taproot yield, has been achieved [1]. However, high yield achievement was usually gained with a penalty of low sucrose content, and vice-versa. Therefore, revealing the regulatory mechanism underlying taproot growth and sucrose accumulation will be of great help for the engineering of new sugar beet cultivars with an optimal balance of taproot yield and sucrose content.

Previously, studies using cDNA amplified fragment length polymorphism (AFLP), ESTs [2, 3] and microarray analyses [4, 5] have been performed to understand the basis of taproot growth and sucrose accumulation at both morphological [6, 7] and physiological levels in sugar beet [8]. During the early development of taproot [5], expressions of genes involved in cell division, as well as those in water and non-electrolyte small molecule transport system, were preferentially expressed. When taproot turned into rapid growth stage, expressions of genes controlled by plant hormones were up-regulated [4]. In addition, genes involved in carbohydrate metabolism were also up-regulated in young sugar beet cells accumulating sucrose [5], and none of those directly involved in sucrose metabolism were up-regulated when taproot was turning into rapid growth stage [4, 5], although the expressions of sucrose synthase genes were positively correlated with the storage root growth rate in radish and sweet potato[9]. The advance in next-generation sequencing technology of transcriptome, as well as the completion of sugar beet genome, has provided an efficient method with spatially higher resolution for the study of the molecular mechanism underlying taproot growth and sucrose accumulation in sugar beet [10].

To explore the molecular regulatory mechanism of storage root development, transcriptomic analyses, a powerful tool for the expression pattern analyses of gene with low abundance of transcripts, have been performed on different crops, such as radish (*Raphanus sativus* L.) [11], carrot (*Daucus carota* L.) [12], sweet potato (*Ipomoea batatas*) [13, 14], cassava (*Manihot esculenta* Crantz) [15], and turnips (*Brassica rapa* L.) [16]. Some candidate genes and regulatory networks involved in storage root development have been identified. However, the precise molecular regulatory mechanism involved in the morphological and physiological phenomena of taproot development in sugar beet is still largely unknown. In this work, the high taproot yield cultivar SD13829 (E-type; E) and high sucrose content cultivar BS02 (Z-type; Z) of sugar beet at five different developmental stages were used to perform comparative transcriptomic analyses. We demonstrate that the weight and sucrose content of taproot rely on its growth strategy, which is controlled by brassinosteroid, auxin, cytokinin, and gibberellin signaling. Among them, an antagonistic expression pattern of brassinosteroid- and auxin-related genes in taproot might play a crucial role for its rapid growth at 82 DAE. We also infer that three GO terms and some members of transcription factors may be involved in the regulation of taproot growth and sucrose accumulation.

## Materials and methods

### Plant materials

The E-type cultivar SD13829 and ST13092 (with low sucrose content) was purchased from Strube CmbH & CO. KG (Sollingen, Germany). Both SD13829 and ST13092 are mono-germy diploid cultivars, with no specific resistance, and a respectively inclined erect and erect plant-type. ST13092 was used for qRT-PCR verification of RNA-seq data only. The Z-type cultivar BS02 and ND0905 (with low yield) was bred by Sugar Beet Physiological Research Institute, Inner Mongolia Agricultural University, Hohhot, China. Both BS02 and ND0905 are pluri-germy diploid cultivars, with no specific resistance, and a respectively erect and inclined erect plant-type. Same as ST13092, ND0905 was used for qRT-PCR verification only, too. All of these cultivars were bred by three-line system hybrid.

**Fig 1:**
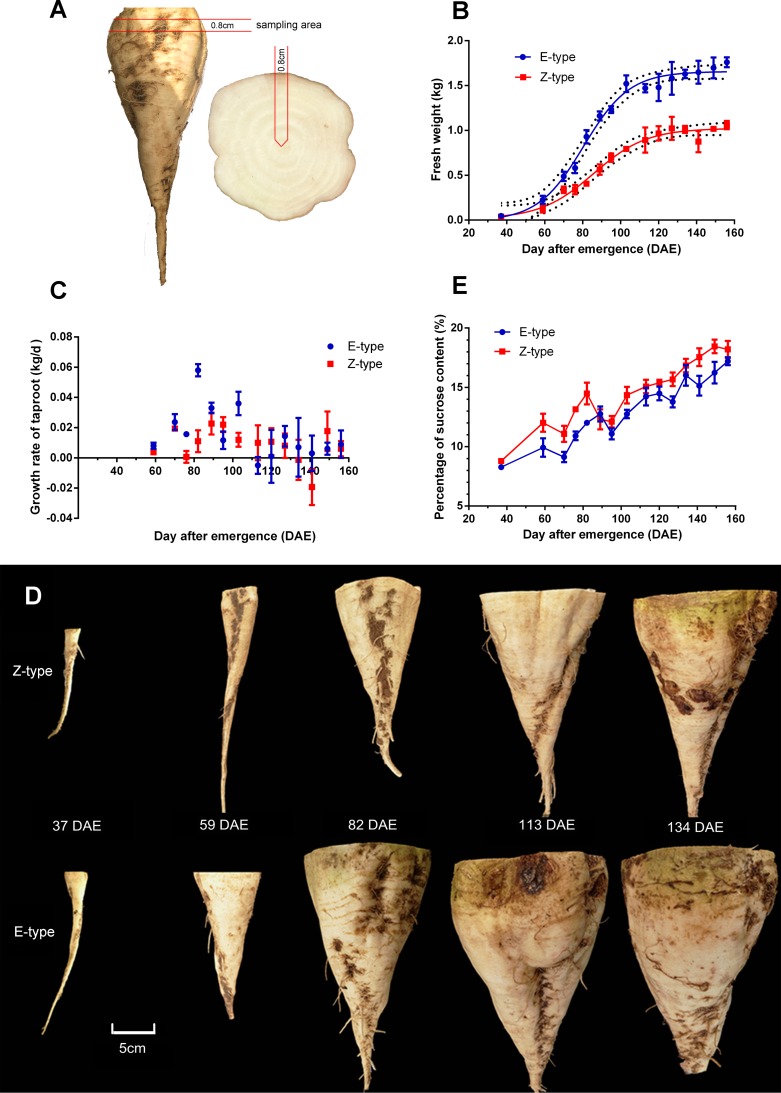
Taproot growth and sucrose accumulation analyses. The E-type cultivar SD13829 and the Z-type cultivar BS02 were grown in field. (A) The sampling area. (B) The growth curve of taproot. (C) The growth rate of taproot. (D) Phenotypes of taproot at different growth stages. (E) Sucrose contents of taproot.

Sugar beet (*Beta vulgaris*) was field planted on the farm of Inner Mongolia Agricultural University at the location of 40°52'54''N, 111°43'53''E. Seeds sowed with 25 cm plant to plant and 50 cm row to row distances on 21 April 2014 emerged on 29 April 2014, and grew under local natural photoperiod. Plant were Irrigated at 21, 47, 71 and 90 DAE (days after emergence), and 450m^3^/hm^2^ water was provided per time. Flusilazole was sprayed at 87, 98, 108 and 118 DAE to prevent cercospora leaf spot disease. In this way, no obvious disease attack was observed. Taproot samples were collected weekly starting at 37 DAE. For each cultivar, 9 taproots (three biological replicates) at each time point were collected. The sampling strategy was based on the number of new leaves. Sucrose content was calculated with a digital handheld refractometer based on the Brix. The average temperature at 37, 59, 82 113 and 134 DAE were 18°C, 24°C, 22°C, 21°C and 16°C, respectively. The sampling strategy of RNA-seq was “overlapping time points with high temporal resolution”, with no biological replicate. The sampling area was shown in Fig 1A.

### Total RNA isolation, cDNA library construction and RNA sequencing

Taproot samples were ground to fine powder using a Frozen Mixer Mill MM400 (RETSCH, Germany). Total RNA was extracted using a Total RNA Isolation System (Takara, Shanghai, China) according to the manufacturer’s instructions. The quality and concentration of total RNA was checked using Nanodrop ND-430 2000, Agilent 2100 Bioanalyzer and a RNA Nano chip device (Agilent, Santa Clara, CA, USA). The quality of RNA of all samples met the requirements of RNA-seq. The isolated mRNA was enriched using oligo-dT beads from 200 ng of total RNA, and then was fragmented. cDNA was synthesized following the manufacturer’s instructions (Invitrogen, Corp., Carlsbad, CA, USA). Fragmented cDNA was treated following the manufacturer’s instructions (Agencourt Bioscience Corporation, Beverly, Massachusetts, USA). cDNA libraries were quantitated by Agilent 2100 bioanalyzer instrument and real-time quantitative PCR, and pair-end sequenced with the HiSeq 2000 System (TruSeq SBS KIT-HS V3, Illumina, 2 × 90 bp)(BGI Tech, Wuhan, China; http://www.bgitechsolutions.com/). Low quality reads and those with adapters or containing more than 10% unknown bases were removed to obtain clean reads.

### Mapping of clean reads and assessment of DEGs

Clean reads were mapped to genome reference (RefBeet-1.1) and gene reference (RefBeet.genes.1302.mrna) [1] using BWA (Burrows-Wheeler Alignment Tool) [17] and Bowtie[18], respectively. To determine which transcripts are isoforms of the same gene, RSEM (RNA-Seq by Expectation Maximization) has been taken as a powerful software package to compute maximum likelihood abundance estimation of each gene and isoform using the Expectation-Maximization (EM) algorithm for its statistical model, including the modeling of paired-end (PE) and variable-length reads, fragment length distributions, and quality scores [19]. The transcripts of genes in both Z-type and E-type were quantified with RSEM, and their expression levels were calculated with the FPKM method [20]. Moreover, differentially expressed genes for pairwise comparisons between each library were identified using a false discovery rate (FDR) ≤ 0.001 [21, 22] and the absolute value of fold change ≥ 2 as the threshold.

### Deep analysis of DEGs

DEGs were blasted to the NCBI non-redundant protein (Nr) database (http://www.ncbi.nlm.nih.gov/) and were annotated using an E-value lower than 10^−5^. Functional annotation was analyzed by the Blast2GO program [23] based on gene ontology terms (GO, http://geneontology.org/). GO enrichment analysis was based on “GO::TermFinder” (http://smd.stanford.edu/help/GOTermFinder/GO_TermFinder_help.shtml/) taking corrected p-value ≤ 0.05, and cluster frequency ≥ 1% as a requirement. The additional requirement, cluster frequency ≥ 1%, was aimed at excluding the terms related with the photosynthesis related to culture effects [24]. The functions of DEGs were also predicted and classed using Kyoto Encyclopedia of Genes and Genomes Pathway (KEGG; http://www.genome.jp/kegg/). For pathway enrichment analysis of DEGs, hypergeometric test value Q≥0.05 was set as the threshold.

### Expression pattern of candidate genes

HemI (Heatmap Illustrator, version 1.0.1; http://hemi.biocuckoo.org/) [25] was used to show expression patterns of candidate genes. Z score transformation [26] was used to perform the standardization in heat maps, which were calculated by subtracting the mean FPKM of all the “main *Beta vulgaris* orthologs” in all of the ten samples, and dividing that result by the standard deviation of all the “main *Beta vulgaris* orthologs” in all of the ten samples for each gene, based on the following formula:

$$Zscore=\left(FPKM_{G}-meanFPKM\right)÷S\text{td}.$$

where G is any *Beta vulgaris* orthologous gene from the transcriptome database. The “main *Beta vulgaris* orthologs” include DEGs or genes with FPKM ≥1 in any library.

### Validation of RNA-Seq by qRT-PCR

Total RNA was treated with DNaseⅠ, and first-strand cDNA was generated using Oligo(dT) (Takara, Japan). Quantitative real-time PCR was performed using LightCycler^®^ 480 (Roche, Switzerland) in a total volume of 20 μl based on iTaq^™^ Universal SYBR^®^ Green Supermix. The condition of reaction was 95°C for 3 min, followed by 40 cycles of 94°C for 10 s, 55°C for 10 s, 72°C for 30 s. All reactions were performed in biological triplicates, and the results were expressed relative to the expression levels of *β-actin* or *GADPH* in each sample by using the 2^-ΔΔCt^ method [27]. Primers were designed using online software (http://www.ncbi.nlm.nih.gov/tools /primer-blast/) with the requirement that PCR products should span an exon-exon junction as possible in order to ignore DNA pollution and alternative splicing.

## Results

### Taproot growth and sucrose accumulation

As a first step to understand the developmental processes of sugar beet taproot, the growth traits of both Z-type and E-type, especially the growth and sucrose content of taproot, were compared. The taproot of both cultivars grew slowly before 59 DAE, but turned into rapid growth stage after this time point until 113 DAE (Fig 1B and 1C). Due to the faster growth rate, the weight of taproot of the E-type cultivar SD13829 was remarkably bigger than that of the Z-type cultivar BS02 throughout almost the whole growing period (Fig 1B and 1D). However, the sucrose content in Z-type was higher than in E-type (Fig 1E). The lower sucrose content in the E-type cultivar SD13829 could be due to the greater taproot weight, which has diluted the sucrose content, and the faster taproot growth which has used up more energy by hydrolyzing sucrose during the growth.

### RNA-Seq libraries and clean read mapping

In order to understand the molecular mechanism involved in the taproot growth and sucrose accumulation in sugar beet, we collected the taproot samples of BS02 (Z-type) and SD13829 (E-type) at 37 DAE (the initial stage of taproot growth), 59 DAE (the transition stage before the rapid growth of taproot), 82 DAE (the rapid growth stage of taproot), 113 DAE (the transition stage before sucrose accumulation), and 134 DAE (the sluggish growth stage of taproot with still active sucrose accumulation), based on their growth and sucrose accumulation. The sampling area for RNA-seq was shown Fig 1A. Different from the tangential width of sampling area at other development stages (0.8cm), the tangential width of sampling area at 37 DAE was 0.3cm. Ten cDNA libraries were constructed for subsequent RNA-Seq analyses. The RNA-Seq reads of these libraries obtained from Illumina HiSeq^™^ 2000 were shown in Table 1. Clean read data were also deposited in the Sequence Read Archive (SRA) of the National Center for Biotechnology Information (NCBI) with the accession number of SRP090408. Clean reads, which count over 97% of the raw reads, were mapped onto a reference gene database (http://bvseq.molgen.mpg.de/Genome/Download/RefBeet-1.1/RefBeet.genes.1302.mrna.fa) including 27,421 protein-coding genes supported by mRNA evidence and filtered for transposable elements [1]. The mapped clean read percentages ranged from 81.75% (library Z-134) to 85.03% (library E-37). Perfectly matched clean read percentages ranged from 56.04% (library Z-134) to 58.95% (library E-37), whereas uniquely matched clean read percentages ranged from 69.77% (library Z-37) to 73.17% (library E-37).

**Table 1:** Summary of mapping results.

Sample Name	Total Reads	Total BasePairs	Total Mapped Reads	Perfect Match	Mismatch	Unique Match	Multi- position Match	Total Unmapped Reads
**Z-37**	55,436,762 (100.00%)	4,989,308,580 (100.00%)	46,676,080 (84.20%)	32,186,180 (58.06%)	14,489,900 (26.14%)	38,680,862 (69.77%)	7,995,218 (14.42%)	8,760,680 (15.80%)
**Z-59**	50,575,736 (100.00%)	4,551,816,240 (100.00%)	41,856,308 (82.76%)	28,401,569 (56.16%)	13,454,739 (26.60%)	35,980,490 (71.14%)	5,875,818 (11.62%)	8,719,426 (17.24%)
**Z-82**	50,438,146 (100.00%)	4,539,433,140 (100.00%)	41,474,712 (82.23%)	28,625,899 (56.75%)	12,848,813 (25.47%)	35,713,530 (70.81%)	5,761,182 (11.42%)	8,963,432 (17.77%)
**Z-113**	51,007,456 (100.00%)	4,590,671,040 (100.00%)	42,192,148 (82.72%)	29,283,962 (57.41%)	12,908,186 (25.31%)	35,899,510 (70.38%)	6,292,638 (12.34%)	8,815,306 (17.28%)
**Z-134**	50,745,002 (100.00%)	4,567,050,180 (100.00%)	41,481,510 (81.75%)	28,437,037 (56.04%)	13,044,473 (25.71%)	35,642,594 (70.24%)	5,838,916 (11.51%)	9,263,490 (18.25%)
**E-37**	50,973,422 (100.00%)	4,587,607,980 (100.00%)	43,344,256 (85.03%)	30,048,993 (58.95%)	13,295,263 (26.08%)	37,298,988 (73.17%)	6,045,268 (11.86%)	7,629,164 (14.97%)
**E-59**	50,920,054 (100.00%)	4,582,804,860 (100.00%)	42,166,760 (82.81%)	29,711,488 (58.35%)	12,455,272 (24.46%)	36,431,802 (71.55%)	5,734,958 (11.26%)	8,753,292 (17.19%)
**E-82**	50,892,430 (100.00%)	4,580,318,700 (100.00%)	42,327,024 (83.17%)	29,509,676 (57.98%)	12,817,348 (25.19%)	36,399,996 (71.52%)	5,927,028 (11.65%)	8,565,404 (16.83%)
**E-113**	50,896,922 (100.00%)	4,580,722,980 (100.00%)	42,252,042 (83.01%)	29,836,824 (58.62%)	12,415,218 (24.39%)	36,153,506 (71.03%)	6,098,536 (11.98%)	8,644,878 (16.99%)
**E-134**	50,999,590 (100.00%)	4,589,963,100 (100.00%)	42,225,672 (82.80%)	29,597,359 (58.03%)	12,628,313 (24.76%)	36,065,036 (70.72%)	6,160,636 (12.08%)	8,773,916 (17.20%)

### Analyses of co-expressed and specifically expressed genes

**Fig 2:**
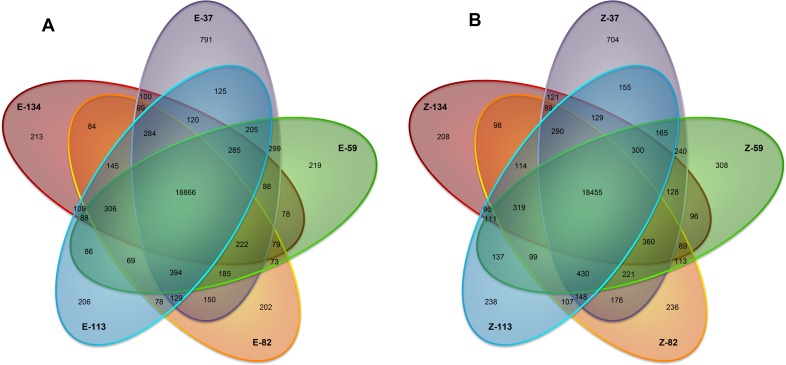
Venn diagrams of co-expressed and specifically expressed genes at five growth stages of taproot. (A) The Venn diagram of E-type. (B) The Venn diagram of Z-type.

We compared the expression levels of genes in both Z-type and E-type. At the five growth stages of 37 DAE, 59 DAE, 82 DAE, 113 DAE and 134 DAE, a total number of 18455 and 18866 genes were respectively co-expressed in Z-type and E-type. The numbers for specifically expressed genes were 704 in Z-type and 791 in E-type at 37 DAE, 308 in Z-type and 219 in E-type at 59 DAE, and 236 in Z-type and 202 in E-type at 82 DAE (Fig 2A and 2B). From 59 to 134 DAE, the numbers of stage-specific genes were relatively constant in both cultivars.

### Screening of differentially expressed genes

**Fig 3:**
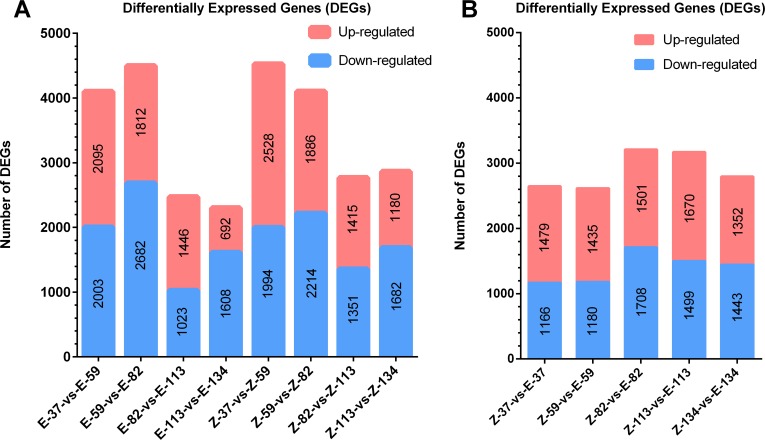
The numbers of differentially expressed genes (DEGs). DEGs obtained in each paired comparison were shown. (A) DEGs obtained in each stepwise comparison. (B) DEGs obtained in each genotype comparison.

The numbers of DEGs from 8 stepwise comparisons, including E-37-vs-E-59, E-59-vs-E-82, E-82-vs-E-113, E-113vs-E-134, Z-37-vs-Z-59, Z-59-vs-Z-82, Z-82-vs-Z-113 and Z-113vs-Z-134, were marked on the top of histogram (Fig 3A). Fewer DEGs were found in the comparisons of Z-82-vs-Z-113, Z-113-vs-Z-134, E-82-vs-E-113 and E-113-vs-E-134, than in Z-37-vs-Z-59, Z-59-vs-Z-82, E-37-vs-E-59 and E-59-vs-E-82, suggesting that gene expression changes have slowed down in the later developmental stages of taproot (Fig 3A).

**Fig 4:**
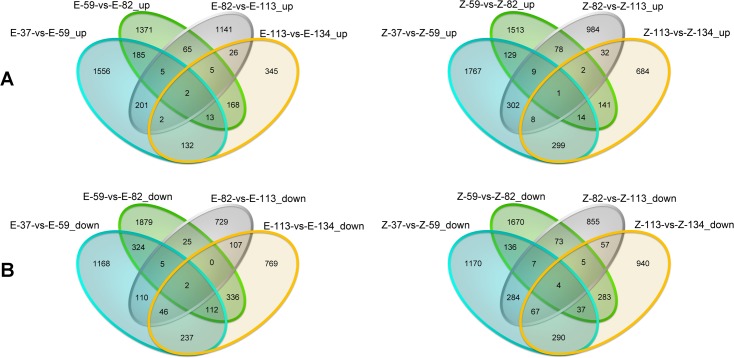
Venn diagrams to show the concurrently and specifically expressed DEGs in different comparison. (A) Significantly up-regulated genes identified at the growth stages of 59 DAE, 82 DAE, 113 DAE and 134 DAE. (B) Significantly down-regulated genes identified at the growth stages of 59 DAE, 82 DAE, 113 DAE and 134 DAE. The numbers of concurrently and specifically expressed DEGs at each development stages were respectively marked in the overlapped and non-overlapped regions.

From the eight comparisons shown in Fig 4, a total number of 1556 and 1168 genes were specifically up- and down-regulated in E-37-vs-E-59, respectively. The numbers of specifically up- and down-regulated genes were 1371 and 1879 in E-59-vs-E-82, 1141 and 729 in E-82-vs-E-113, 345 and 769 in E-113-vs-E-134, 1767 and 1170 in Z-37-vs-Z-59, 1513 and 1670 in Z-59-vs-Z-82, 984 and 855 in Z-82-vs-Z-113, and 684 and 940 in Z-113-vs-Z-134 (Fig 4A and 4B). Compared to other comparisons, more concurrently up-regulated genes were found in the comparisons of E-37-vs-E-59 and E-59-vs-E-82, with a total number of 185 genes, and in the comparisons of Z-37-vs-Z-59 and Z-59-vs-Z-82, with a total number of 129 genes.

### Gene ontology analyses of DEGs

**Fig 5:**
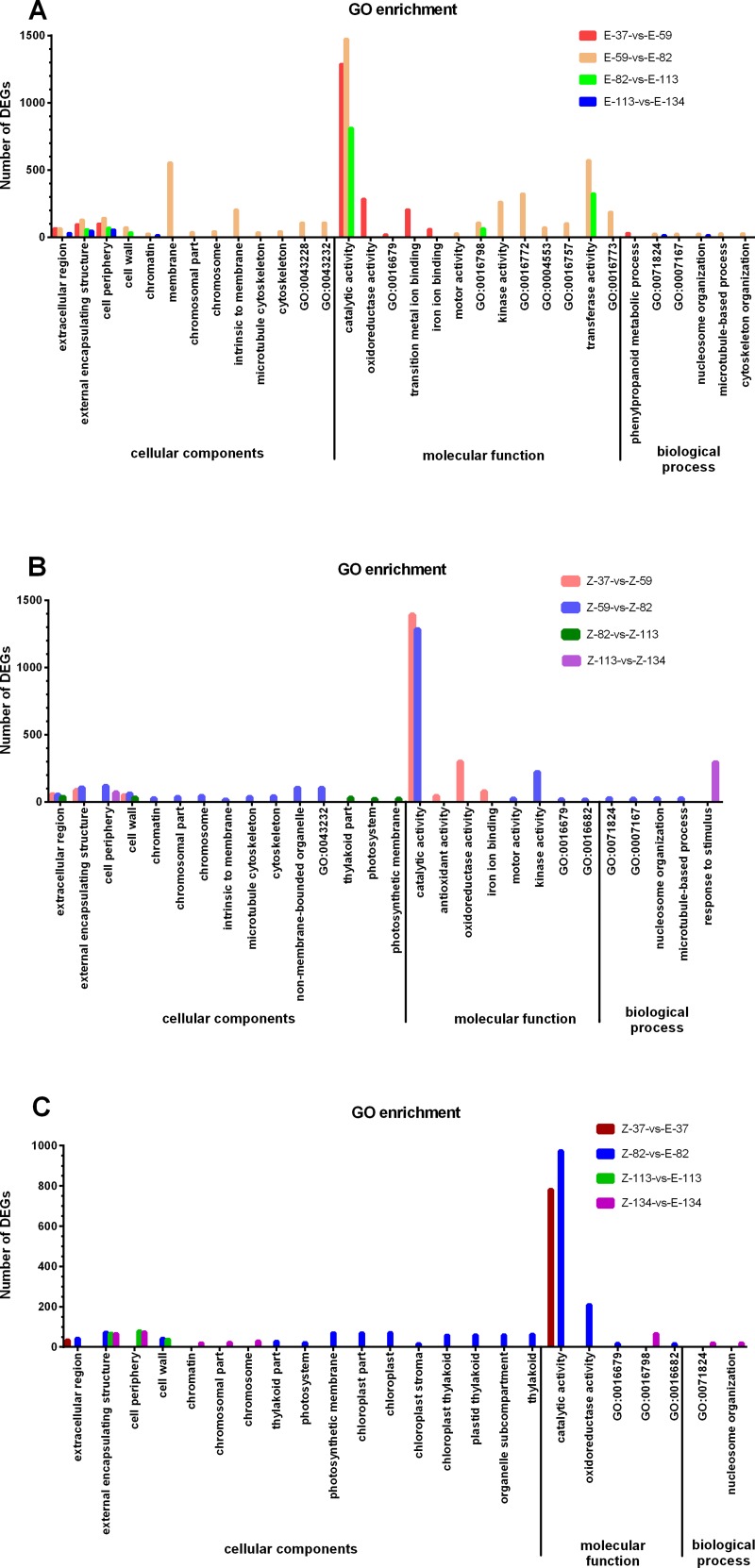
GO enrichment analyses. (A) The enriched terms of DEGs gained from the comparisons of E-37-vs-E-59, E-59-vs-E-82, E-82-vs-E-113 and E-113vs-E-134. (B) The enriched terms of DEGs gained from the comparisons of Z-37-vs-Z-59, Z-59-vs-Z-82, Z-82-vs-Z-113 and Z-113vs-Z-134. (C) The enriched terms of DEGs gained from the comparisons of Z-37-vs-E-37, Z-59-vs-E-59, Z-82-vs-E-82, Z-113-vs-E-113 and Z-134-vs-E-134. No enriched term in the comparison of Z-59-vs-E-59 was identified. The names of GO terms were replaced with GO accession numbers and shown in S8 Table.

GO is composed of three ontologies: cellular component, molecular function, and biological process. Gene function was defined with GO ID gained at gene annotation. Usually, a significantly enriched GO term may be related to a biological problem. In the comparison of E-37-vs-E-59, a total number of 1378, 1760 and 1457 DEGs were respectively assigned to cellular component, molecular function and biological process (Table 2). More detailed results were shown in S1 Table. Taking corrected p-value ≤ 0.05 and cluster frequency ≥ 1% as a threshold, the GO terms, which showed significant enrichment in the ontology of cellular component, molecular function and biological process, respectively, in each comparison, were provided in Fig 5.

**Table 2:** GO enrichment analyses of DEGs.

comparison	cellular component	molecular function	biological process
**E-37-vs-E-59**	1378	1760	1457
**E-59-vs-E-82**	1630	2013	1695
**E-82-vs-E-113**	852	1088	891
**E-113-vs-E-134**	675	917	758
**Z-37-vs-Z-59**	1457	1924	1554
**Z-59-vs-Z-82**	1394	1782	1437
**Z-82-vs-Z-113**	842	1116	899
**Z-113-vs-Z-134**	900	1142	934
**Z-37-vs-E-37**	723	1044	805
**Z-59-vs-E-59**	748	1006	784
**Z-82-vs-E-82**	1007	1341	1096
**Z-113-vs-E-113**	981	224	1000
**Z-134-vs-E-134**	836	1112	911

In the comparison of E-59-vs-E-82, significantly enriched DEGs in the cellular component GO term were external encapsulating structure, cell periphery, cell wall, extracellular region, chromatin, membrane, chromosomal part, chromosome, intrinsic to membrane, microtubule cytoskeleton, cytoskeleton, external encapsulating structure part, non-membrane-bounded organelle, and intracellular non-membrane-bounded organelle. Significantly enriched DEGs in the molecular function GO term were motor activity, catalytic activity, hydrolase activity, acting on glycosyl bonds, kinase activity, transferase activity, transferring phosphorus-containing groups, hydrolase activity, hydrolyzing O-glycosyl compounds, transferase activity, transferring glycosyl groups, transferase activity, phosphotransferase activity, and alcohol group as acceptor. Significantly enriched DEGs in the biological process GO terms were protein-DNA complex subunit organization, enzyme linked receptor protein signaling pathway, nucleosome organization, microtubule-based process, and cytoskeleton organization. Results for the GO significantce enrichment analyses of DEGs in other paired comparisons were shown in Fig 5 and S1 Table. Based on the above analyses, the most significantly enriched GO terms were identified at 82 DAE, the most rapidly expanding stage of taproot.

### KEGG pathway enrichment analyses of DEGs

KEGG pathway enrichment analyses can provide the insight into how taproot growth and sucrose metabolism are regulated. Usually, DEGs in the same pathway exercise their biological functions together. As shown in Table 3 and S2 Table, in the comparison of E-37-vs-E-59, a total number of 2185 DEGs were mapped to the KEGG plant database, and 1674 DEGs of them were significantly enriched in 17 different KEGG pathways. For the rapid taproot growth stage (from 59 to 82 DAE), these results imply a very active metabolism, cell division and cell growth differentiation in the taproot of sugar beet. Interestingly, more enriched KEGG pathways were observed in the E-type cultivar SD13829 than in the high sucrose but low yield cultivar BS02. Detailed results of each comparison were shown in S2 Table. For DEGs in plant hormone signal transduction pathway of the comparison of E-59-vs-E-82, which is critical for the rapid growth of taproot, the xyloglucan: xyloglucosyl transferase (TCH4; EC: 2.4.1.207) encoding gene *Bv_51330_psrc*.*t1* showed the highest up-regulation (except the specifically expressed genes). Whereas *Bv3_067020_oenm*.*t1* that encodes the protein brassinosteroid insensitive 1 (EC: 2.7.10.1; 2.7.11.1) showed the highest down-regulation. More detailed results were provided in S3 Table.

**Table 3:** Pathway enrichment analyses of DEGs.

Comparison	Number of DEGs mapped to KEGG pathway	Number of DEGs belong to the significantly enriched pathway	Number of significantly enriched pathway
**E-37-vs-E-59**	2185	1674	17
**E-59-vs-E-82**	2419	1255	16
**E-82-vs-E-113**	1328	488	6
**E-113-vs-E-134**	1178	290	3
**Z-37-vs-Z-59**	2371	1911	19
**Z-59-vs-Z-82**	2217	431	3
**Z-82-vs-Z-113**	1408	1118	13
**Z-113-vs-Z-134**	1471	404	7
**Z-37-vs-E-37**	1332	970	12
**Z-59-vs-E-59**	1271	1063	18
**Z-82-vs-E-82**	1628	1111	9
**Z-113-vs-E-113**	1567	284	5
**Z-134-vs-E-134**	1404	1015	13

At the growth stage from 113 to 134 DAE, taproot growth became sluggish but sucrose accumulation was still active. Coincidently, the Z-type cultivar BS02 showed more significantly enriched KEGG pathways than did the E-type cultivar SD13829. More information was given in S2 Table.

### Candidate genes involved in plant hormone biosynthesis and metabolism

**Fig 6:**
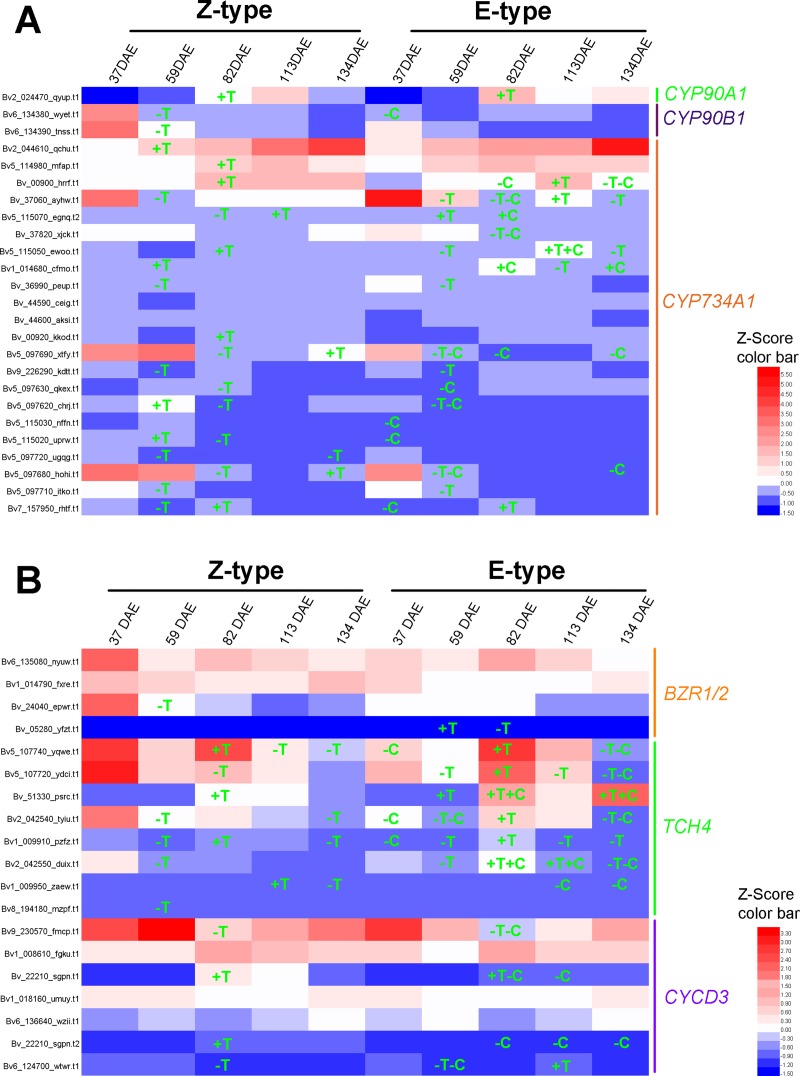
Expression pattern of genes involved in the brassinosteroid signaling. (A) Orthologues of genes encoding the key enzymes for the biosynthesis and metabolism of brassinosteroid. (B) Orthologues of genes encoding the components in the signal transduction pathways of brassinosteroid. Z score transformation [26] was calculated by subtracting the mean FPKM of all the “main *Beta vulgaris* orthologs” in all of the ten samples, and dividing that result by the standard deviation based on all the “main *Beta vulgaris* orthologs” in all of the ten samples for each gene. The “main *Beta vulgaris* orthologs” included the DEGs or the genes with FPKM ≥1 in any library. Gene IDs were marked on the left. +, up-regulated; -, down-regulated; T, up- and down-regulated compared the previous growth stage; C, E-type compared with Z-type.

**Fig 7:**
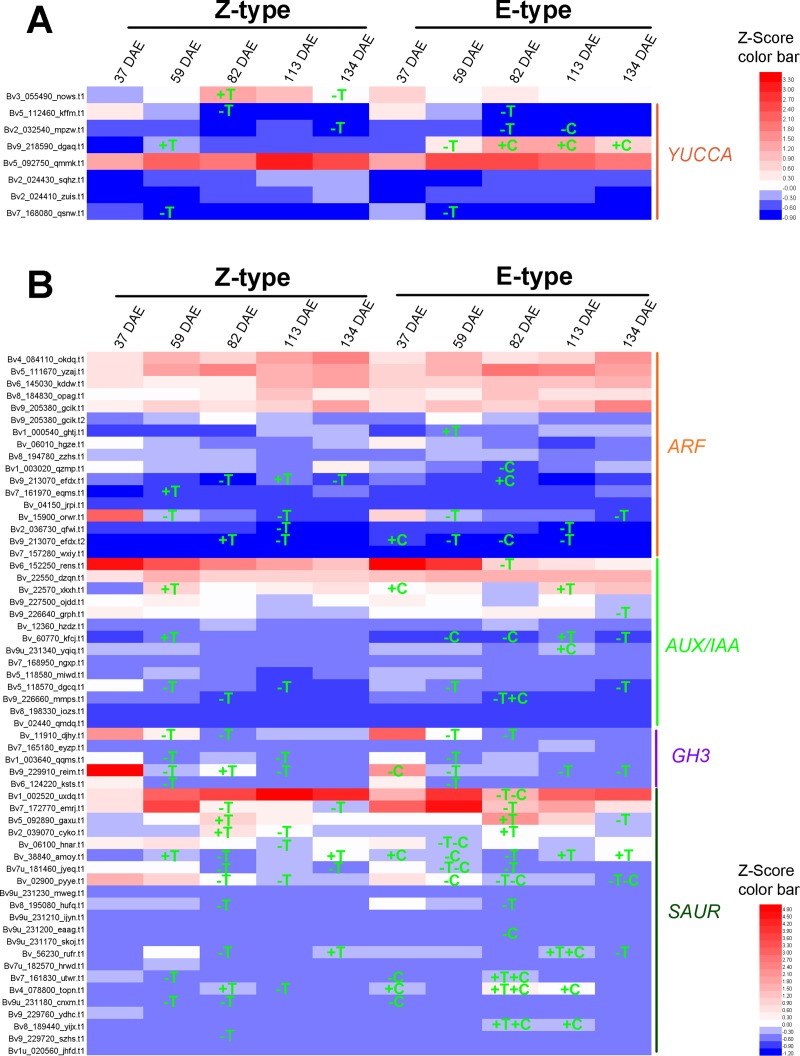
Expression pattern of genes involved in auxin signaling. (A) Orthologues of genes encoding the key enzyme for the biosynthesis of auxin. (B) Orthologues of genes encoding the components in the signal transduction pathways of auxin. Gene IDs were marked on the left. +, up-regulated; -, down-regulated; T, up- and down-regulated compared to the previous growth stage; C, E-type compared with Z-type.

**Fig 8:**
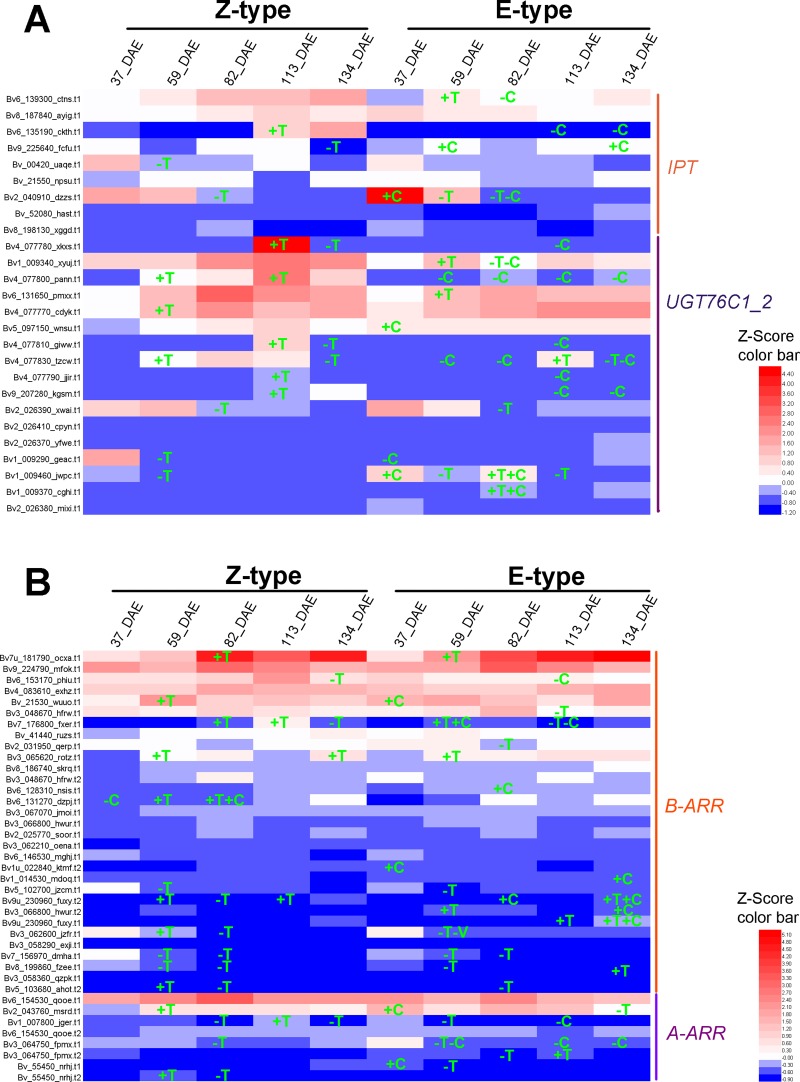
Expression pattern of genes involved in cytokinin signaling. (A) Orthologues of genes encoding the key enzymes for the biosynthesis and metabolism of cytokinin. (B) Orthologues of genes encoding the components in the signal transduction pathways of cytokinin. Gene IDs were marked on the left. +, up-regulated; -, down-regulated; T, up- and down-regulated compared to the previous growth stage; C, E-type compared with Z-type.

**Fig 9:**
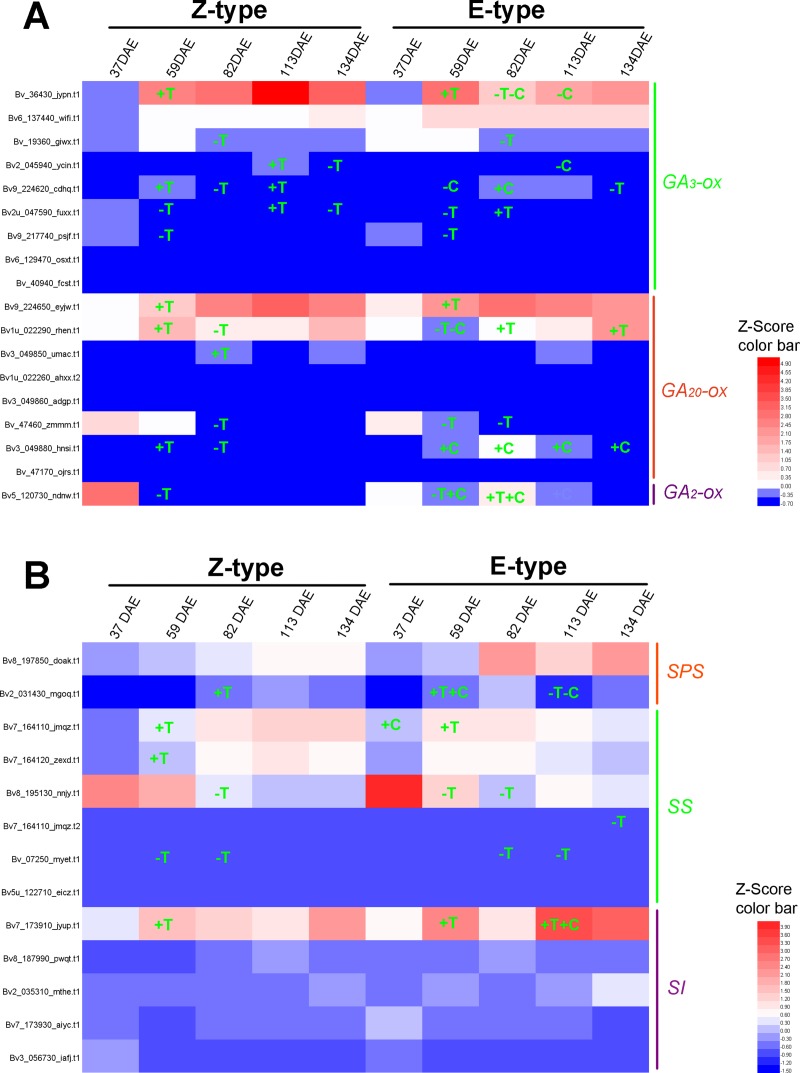
Expression pattern of genes involved in the biosynthesis and metabolism of gibberellin and sucrose. (A) Orthologues of genes encoding the key enzymes for the biosynthesis and metabolism of gibberellin. (B) Genes involved in sucrose biosynthesis and metabolism. Gene IDs were marked on the left. +, up-regulated; -, down-regulated; T, up- and down-regulated compared to the previous growth stage; C, E-type compared with Z-type.

In this study, 2 of 4 *Beta vulgaris CYP90B1* (K09587) orthologs [28] and 1 of 2 *Beta vulgaris CYP90A1* (K09588) orthologs [29] which encode the rate-limiting enzymes for brassinosteroid biosynthesis were identified as DEGs in one or more paired comparisons (Fig 6A, S4 Table). DEGs, 5 of 16 *YUCCA* (K11816) for IAA [30], 5 of 13 *IPT* (K10760) for zeatin [31], 6 of 13 *GA*_*3*_*-ox* (K04124) [32] and 5 of 11 *GA*_*20*_*-ox* (K05282) [33] for GA, which encode the rate-limiting enzymes for other hormone biosynthesis, were also identified (Figs 7A, 8A and 9A, S4 Table). Furthermore, 20 of the 29 *Beta vulgaris CYP734A1* (K15639) orthologues which encode the PHYB activation tagged suppressor 1 involved in brassinosteroid metabolism [34] were identified as DEGs (Fig 6A and S4 Table). DEGs, 14 of 24 *UGT76C1_2* (K13493) for zeatin [35] and 1 of 1 *GA*_*2*_*-ox* (K04125) for GA [36] involved in other hormone metabolism, were also identified (Figs 8A and 9A, S4 Table).

### DEGs in plant hormone signal transduction pathway

In the analyzed thirteen comparisons, a total number of 98 to 170 DEGs were enriched in plant hormone transduction pathways (S2 Table). In brassinosteroid signal transduction pathway, 2 of 5 *Beta vulgaris* brassinosteroid resistant 1/2 (*BZR1*/*2*) orthologues, 8 of 10 *Beta vulgaris TCH4* orthologues, and 4 of 10 *Beta vulgaris CYCD3* orthologues were identified as DEGs (Fig 6B, S5 Table) [37, 38]. In the auxin signal transduction pathway, 7 of 17 auxin response factor (*ARF*) orthologues, 7 of 16 auxin-responsive gene IAA (*AUX/IAA*) orthologues, 4 of 5 auxin responsive GH3 family genes (*GH3*), and 17 of 38 SAUR family genes (*SAUR)* were identified as DEGs (Fig 7B, S5 Table). In the cytokinin signal transduction pathway, 20 of 38 *Beta vulgaris B-ARR* orthologues encoding two-component response regulator ARR-B family transcription factor [39], and 6 of 9 *Beta vulgaris A-ARR* orthologues of the two-component response regulator ARR-A family which negatively regulate cytokinin signaling, were identified as DEGs (Fig 8B, S5 Table) [40].

### Genes involved in sucrose metabolism

In order to understand the molecular mechanism of sucrose accumulation in sugar beet taproot, we analyzed DEGs involved in sucrose metabolism. We found that taproot sugar content increased during the early developmental stages (Fig 1E), but decreased at the growth stage from 82 to 95 DAE. We further compared the expressions of *SPS* (sucrose phosphate synthase; EC2.4.1.14), *SS* (sucrose synthase; EC 2.4.1.13) and *SI* (sucrose invertase; EC 3.2.1.26), the major candidate genes in the sucrose metabolic pathway, and found that 1 of 2 *Beta vulgaris SPS* orthologues, 5 of 7 *Beta vulgaris SS* orthologues and 1 of 9 *Beta vulgaris SI* orthologues were identified as DEGs in one or more comparisons (Fig 9B, S6 Table). The expression level of *SPS* orthologue, *Bv2_031430_mgoq*.*t1*, was up-regulated 2.93 folds at 82 DAE in Z-type, whereas the fold changes in E-type at 59 and 113 DAE were 4.37 and 0.24, respectively. The expression levels of these 5 *SS* orthologues in stepwise comparisons were up-regulated from 2.10 to 4.37 folds, and were down-regulated from 0.44 to 0.23 fold. The expression level of *SI* orthologue, *Bv7_173910_jyup*.*t1*, was up-regulated 2.17 folds at 59 DAE in Z-type, whereas the fold changes in E-type at 59 and 113 DAE were respectively 2.16 and 2.28.

### The analyses of DEGs in genotype comparisons

In addition to the stepwise comparisons, the numbers of DEGs from 5 genotype comparisons, including Z-37-vs-E-37, Z-59-vs-E-59, Z-82-vs-E-82, Z-113-vs-E-113 and Z-134-vs-E-134, were marked on the top of histogram (Fig 3B). At the active growth stages of 82 and 113 DAEs, more DEGs were identified (Fig 3B).

The DEGs of significantly enriched GO term are usually related to a biological problem. The numbers of significantly enriched GO terms in the comparison of Z-82-vs-E-82 and Z-113-vs-E-113 were respectively 17 and 3 (Fig 5C). KEGG pathway enrichment analyses can provide the insight into why taproot weight and sucrose content are different in E-type and Z-type cultivars. A total number of 9 and 5 KEGG pathways were significantly enriched in the comparison of Z-82-vs-E-82 and Z-113-vs-E-113 respectively (S2 Table). In addition, pant hormone signal transduction pathway, including 107 DEGs, was enriched in the comparison of Z-82-vs-E-82, with a Q value of 0.05437378 (S2 Table).

In order to gain insight into how pant hormone signal transduction pathway drove physiological differences between E-type and Z-type, we deeply analyzed the expression level of some candidate genes (Figs 6–9). These candidate genes included genes encoding the rate-limiting enzymes for plant hormone biosynthesis and metabolism, DEGs in plant hormone signal transduction pathway and genes involved in sucrose metabolism. The expression pattern of these candidate genes was shown in Figs 6–9.

### Differentially expressed transcription factors (TFs) at 82 DAE

In this study, members of various transcription factor (TF) families were identified. In the comparisons of Z-59-vs-Z-82, E-59-vs-E-82 and Z-82-vs-E-82, a total number of 60, 73, and 42 TF encoding genes were identified as DEGs, respectively. Among them, 32 out of 60, 42 out of 73, and 23 out of 42 genes were up-regulated. In addition, 22 genes were concurrently up-regulated in the comparison of Z-59-vs-Z-82 and E-59-vs-E-82, 11 genes were concurrently up-regulated in the comparisons of E-59-vs-E-82 and Z-82-vs-E-82, and 6 genes were concurrently up-regulated in the above three comparisons (Table 4).

**Table 4:** Commonly up-regulated TF genes in the comparisons of Z-59-vs-Z-82, E-59-vs-E-82 and Z-82-vs-E-82.

Gene ID	TF family	log_2_Ratio (Z-59-vs-Z-82)	log_2_Ratio (E-59-vs-E-82)	log_2_Ratio (Z-82-vs-E-82)
**Bv_07170_ishy.t1**	zf-HD	9.94	3.72	2.15
**Bv8_193570_hpsu.t1**	C2H2	3.90	3.64	1.01
**Bv9_229380_fkap.t1**	WRKY	3.53	3.37	1.13
**Bv1_012110_zfms.t1**	Dof	1.35	3.29	1.99
**Bv1_012090_yjik.t1**	Dof	2.48	3.16	1.39
**Bv6_130710_cdca.t1**	Dof	1.52	1.30	5.45

### Verification of transcriptomic data

**Fig 10:**
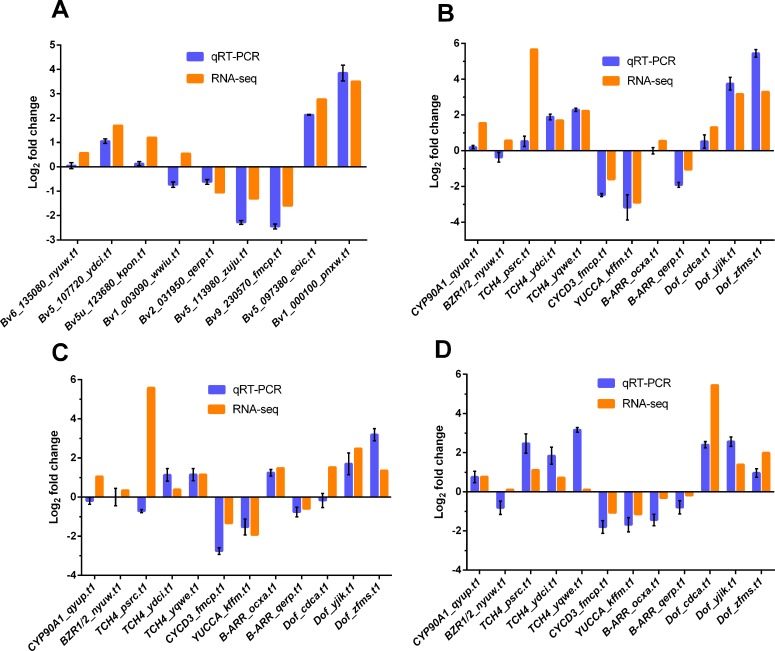
Comparisons of the gene expression as determinated by RNA-Seq and qRT-PCR analyses, respectively. (A) Expression pattern validation of transcriptomic data in SD13829 or BS02 by qRT-PCR. (B) Expression pattern validation of selected genes in another E-type cultivar ST13092. (C) Expression pattern validation of selected genes in another Z-type cultivar ND0905. (D) Comparisons of the selected gene expression in the comparison of Z-82-vs-E-82 determined by RNA-Seq and ND0905-82-vs-ST13092-82 determined by qRT-PCR. Gene ID, abbreviation and primer of target gene were shown in S9 Table.

To verify the validity of transcriptomic data, 9 genes including *Bv6_135080_nyuw*.*t1*, *Bv5_107720_ydci*.*t1*, *Bv5u_123680_kpon*.*t1*, *Bv1_003090_wwiu*.*t1*, *Bv2_031950_qerp*.*t1*, *Bv5_113980_zuju*.*t1*, *Bv9_230570_fmcp*.*t1*, *Bv5_097380_eoic*.*t1* and *Bv1_000100_pnxw*.*t1* were randomly selected for real time RT-PCR (qRT-PCR) analyses. As shown in Fig 10A, the results were consistent with the RNA-Seq data, suggesting that transcriptomic sequencing in this study revealed actual gene expressions during sugar beet taproot development. We also performed qRT-PCR analyses for some crucial genes in another E-type and Z-type cultivar at 59 and 82 DAE (Fig 10B–10D). These crucial genes included 1 orthologue of *CYP90A1*, 1 orthologue of *BZR1/2*, 3 orthologues of *TCH4*, 1 orthologue of *CYCD3*, 1 orthologue of *YUCCA*, 2 orthologues of *B-ARR*, and 3 orthologues of *Dof*.

## Discussion

The development of *Beta vulgaris* taproot includes three interrelated processes: (1) cell division in the secondary meristem rings [6, 41]; (2) turgor-driven cell enlargement along the derivatives of cambiums [5, 6]; and (3) sucrose accumulation in the parenchyma cells [5]. During the development of taproot, the growth and sucrose accumulation in it are two opposite agronomic traits. A better understanding of *Beta vulgaris* taproot development may be achieved through the analyses of the molecular mechanisms underpinning this complex developmental process. Although some genes involved in *Beta vulgaris* taproot growth and sucrose accumulation have been identified by EST sequencing or cDNA microarray technique [2, 4, 5], the precise mechanisms regulating these processes are still not clear. Previously, we appraised the yield and quality of a total of 79 sugar beet cultivars. And two typical E-type cultivars, SD13829 and ST13092, and two typical Z-type cultivars, BS02 and ND0905, were identified. In order to explore the molecular regulatory mechanism of taproot growth and sucrose accumulation, the E-type cultivar SD13829 and Z-type cultivar BS02 of sugar beet were used to perform comparative transcriptomic analyses.

Based on the growth curve of taproot development (Fig 1B, 1C and 1E), which was consistent with previous reports [5, 6], the key developmental stages for taproot growth and sucrose accumulation were 37 DAE, 59 DAE, 82 DAE, 113 DAE and 134 DAE. Taproot sucrose content of SD13829 and BS02 was respectively similar to that of USH20 and SR96 [4]. Among them, the key determining stages for taproot weight were 59 DAE and 82 DAE. We then performed comparative transcriptomic analyses at the above stages. The results of clean read mapping (Table 1) were similar to the data reported in potato, maize and rice [42–44]. Analyses of co-expressed and specifically expressed genes were designed to check the elementary stage (the initial stage before the taproot starts to develop) of taproot development. The numbers of co-expressed genes in two cultivars revealed that there were about 18000 housekeeping genes during all stages, which might be required for the regular growth of taproots. The numbers of specifically expressed genes at 37 DAE for both cultivars were significantly greater than that at other stages (Fig 2A and 2B). Therefore, the elementary stage of taproot development was 37 DAE. The numbers of specifically expressed genes at other stages were relatively constant in both cultivars (Fig 2A and 2B). But the function of the specifically expressed genes in each stage was different from the others. One of the reasons for this constancy is that different specifically expressed genes are required for different growth stages of taproots. More DEGs were identified at 82 and 113 DAEs (Fig 3), suggesting that the molecular regulation of taproot development was more activity at these stages. In every stepwise comparison, the different number of DEGs and of up- and down-regulated genes is relatively similar between E-type and Z-type; however, it seems that the number of genes specifically activated/repressed in that time lapse follows a different pattern for the two genotypes (Fig 4A and 4B). Therefore, the stage-determining genes are more or less the same, but they are regulated at slightly different times in the two sugar beet types. The analyses of concurrently up- and down-regulated genes (Fig 4A and 4B) showed that 59 DAE and 82 DAE were the more active growth stages for the molecular regulation of taproot development. Therefore, we perform further analyses of DEGs at the stages of 59 DAE and 82 DAE.

In the studies on the taproot development of radish and carrot, several key GO terms (such as cell wall, cytoskeleton, and regulation of biological process) have been identified [11, 12, 45]. These GO terms reflected the major physiological events of storage root development. In order to annotate the DEGs in the 8 stepwise comparisons, we performed GO enrichment analyses, and found that three GO terms including cell wall, cytoskeleton and enzyme linked receptor protein signaling pathway (the child term of regulation of biological process) were enriched at 82 DAE when taproot turned into the rapidest growth stage. Among these DEGs, the up-regulated genes at 82 DAE included genes encoding pectinesterase and beta-glucosidase (bglB) involved in cell wall elongation [46], dynein light chain LC8-type, kinesin family member C2/C3 and anaphase-promoting complex subunit 3 that regulate mitosis in transcriptional level [47–50], FERONIA receptor-like kinase (FER) [51], gibberellin-regulated protein 1, protein brassinosteroid insensitive 1 [52, 53], and receptor-like protein kinase HAIKU2 [54, 55] involved in the signal transduction of plant development and the controlling of cell size [56, 57] (S7 Table). These results suggest that DEGs enriched in these three GO terms were responsible for the rapid growth of taproot [13] via the regulation of cell wall elongation, which depends on cell growth, mitosis, and cell wall sensing signal transduction.

KEGG pathway enrichment analyses has been used as a powerful tool to understand the functions of biological systems in plants [58]. Previously, it was reported that plant hormone signal transduction pathway, zeatin biosynthesis pathway and secondary metabolite biosynthesis pathway were associated with taproot development [59], and the starch and sucrose metabolic pathway was also involved in the secondary root thickening in radish [11]. Here, we demonstrate that these genes may regulate taproot growth together. KEGG pathway enrichment analyses of DEGs in the comparisons of E-59-vs-E-82 and Z-59-vs-Z-82 were carried out. In the comparisons of E-59-vs-E-82 and Z-59-vs-Z-82, a total number of 170 and 158 DEGs were significantly enriched in the plant hormone signal transduction pathway, respectively (S2 Table). Therefore, similar to the observations in radish [59], plant hormone signal transduction pathway was involved in the regulation of taproot growth rates from 59 to 82 DAE. However, different from the results in radish [59], the brassinosteroid biosynthesis pathway was enriched in the comparisons of E-37-vs-E-59 and Z-37-vs-Z-59 in our study (S2 Table), suggesting that the growth strategy of sugar beet taproot, which was mainly regulated by brassinosteroid, is different from that of radish. The starch and sucrose metabolism pathway was enriched in the comparisons of E-113-vs-E-134 (S2 Table). But the plant hormone signal transduction pathway was enriched only in the comparison of E-113-vs-E-134 (S2 Table). Therefore, both pathways could be involved in the functional transition from taproot growth to sucrose accumulation at the growth stages from 113 to 134 DAE.

A set of plant hormones (i.e. brassinosteroid, auxin, cytokinin and GA) have been studied in root development in the model plant species such as *Arabidopsis* and rice [24, 60, 61], or non-model species producing storage root such as radish, sweet potato and sugar beet [5, 42, 59]. In taproot with tertiary structure, cellular communication is very important for the coordinating division and growth of cambium cells [24]. Brassinosteroid, which functions as cellular signaling molecular, can improve root cell division and growth in plants [60, 62]. So we further analyzed DEGs involved in brassinosteroid biosynthesis, metabolism and signal transduction during taproot development (Fig 6A and 6B). The expression of *CYP90A1*, encoding the rate-limiting enzyme steroid hydroxylase in brassinosteroid biosynthesis, was up-regulated at 82 DAE compared to that at 59 DAE. Interestingly, only 1 in E-type, but 5 in Z-type, of *CYP734A1* orthologues, which is also involved in the regulation of brassinosteroid metabolism, were up-regulated at 82 DAE [63]. Therefore, the higher brassinosteroid content (data not shown) in the E-type cultivar SD13829, which was related to the faster growth of taproot, may be due to the relatively lower metabolic activity in it [60].

Brassinosteroid signal transduction also plays a crucial role in taproot development [12]. In higher plants, the activity of BZR1/2, the transcription factor of brassinosteroid signal transduction pathway [64], is mainly regulated by dephosphorylation at post transcription level [65]. At 82 DAE, only 1 of *BZR1/2* orthologue was down-regulated in the E-type (Fig 6B). The expression level of *TCH4*, a xyloglucan:xyloglucosyl transferase encoding gene, showed similar expression pattern to the taproot growth rate in both E-type and Z-type (Fig 6B). We postulated that *TCH4* could also be involved in the taproot growth of sugar beet. This postulation is also partially supported by previous studies in sugar beet [5] and radish [66].

In *Arabidopsis*, it has been well documented that both brassinosteroid and auxin signaling work together to regulate root development [60, 62, 67, 68]. At the growth stages from 59 DAE to 82 DAE, of the three *YUCCA* orthologues, one was down-regulated in both E-type and Z-type, one was up-regulated in the Z-type, and the other one was down-regulated in the E-type (Fig 7A). The expression of important components in auxin signal transduction pathway (*ARF*, *AUX/IAA*, *GH3* and *SUAR*) followed an opposite pattern with the rate of taproot growth (Fig 7B). Similar results were also observed in the roots of *Arabidopsis* [60]. In plants, optimal root growth requires a balance between the antagonistic actions of brassinosteroid and auxin [60, 67]. Our results imply that in opposite to their synergistic functions in shoots, brassinosteroid and auxin work antagonistically in taproot to regulate the spatiotemporal balance of secondary cambium cell dynamics. Therefore, the functional integration of auxin and brassinosteroid is important for optimal taproot growth.

In radish, in addition to brassinosteroid and auxin, cytokinin is also an important regulator which drives the radial storage root growth via controlling cell proliferation in the secondary cambium [69]. But in sugar beet, it is not clear whether cytokinin signalling regulates taproot growth. As shown in Fig 8B, a total number of 9 *B-ARR* orthologues were up-regulated at the growth stages from 37 DAE to 59 DAE. A total number of 6 *B-ARR* orthologues were down-regulated at the growth stage of 82 DAE (Fig 8B). Therefore, when taproot turned into rapid growth stages from 37 to 82 DAE, *B-ARR* worked actively, with the highest activity at 59 DAE. Similar results were also reported in radish [59]. Based on these analyses, we conclude that cytokinin signal transduction pathway also plays a critical role in taproot growth.

In sugar beet, gibberellin activity was well correlated with the period of rapid cell enlargement at the temporal scale [6]. *GA*_*20*_*-ox* was preferentially expressed during late development, while *GA*_*2*_*-ox* was preferentially expressed during early development [5]. In our study, similar expression patterns of them were observed, with an earlier predominant expression of *GA*_*20*_*-ox* in both E-type and Z-type, and *GA*_*2*_*-ox* in the Z-type (Fig 9A). The expression of *GA*_*3*_*-ox* followed a similar pattern to that of *GA*_*20*_*-ox*. These results demonstrate that gibberellin functions in cell enlargement during the later development since 59 DAE.

In addition to taproot yield, taproot sucrose content is another economically important character. We analyzed the expressions of sucrose metabolic enzymes, including SPS, SS and SI, which not only regulate phloem unloading and import of sucrose into taproots [70–72], but also provide energy and substance for cell growth [73, 74]. As shown in Fig 9B, 1 *SPS* orthologue was up-regulated following the transition of rapid growth stage in both cultivars. The expression of *Bv7_173910_jyup*.*t1* encoding a beta-fructofuranosidase (cell-wall invertase-like) followed a similar pattern with an up-regulation at 59 DAE. These results were different from the previous report that neither *SPS* nor *SI* orthologues were differentially expressed [5]. One explanation is that DEGs were identified by rational sampling and advanced sequence technology in our study.

In previous studies with radish and sweet potato, *SS* was the most actively expressed gene compared to *SPS* and *SI* in sucrose metabolism, and was related to storage root development [9, 66]. Similar to the results in radish [11], 2 of 3 main *SS* orthologues, which showed much higher expression levels than the other *SS* family members, were up-regulated at 59 DAE (Fig 9B). The other one showed a higher expression level at the growth stages of 37 and 59 DAE compared to the later developmental stages (Fig 9B), which was consistent with the previous microarray analyses in sugar beet taproot development [5]. In addition, *Bv7_164110_jmqz*.*t1* and *Bv7_164110_jmqz*.*t2*, the product of alternative splicing, showed different expression patterns. The expression level of *Bv7_164110_jmqz*.*t1* was up-regulated 2.10 and 3.32 folds in both E-type and Z-type at 59 DAE, respectively, and was up-regulated 2.25 folds in the E-type than that in the Z-type at 37 DAE. But, the expression of *Bv7_164110_jmqz*.*t2* was only down-regulated 0.37 fold in the Z-type at 134 DAE. These results suggest that *SS* could be involved in the later development stages of taproot growth, and the early development stages of sucrose accumulation. The sharp decrease of sucrose content from 82 to 95 DAE could be due to the faster cell growth and sucrose metabolism.

Based on the molecular regulatory mechanism of taproot growth, we analyzed the DEGs in the genotype comparisons to gain insight into the type-determining molecular mechanism. As discussion above, DEGs enriched in the GO terms of cell wall, cytoskeleton and enzyme linked receptor protein signaling pathway were related to the rapid growth of taproot. We found that the cell wall GO term was also enriched in the comparison of Z-82-vs-E-82. In the comparison of Z-113-vs-E-113, three GO terms, cell wall, cell periphery and external encapsulating structure, were significantly enriched. Therefore, DEGs enriched in the cell wall GO term could be responsible for the difference of taproot growth rate that occurred between both E-type and Z-type at 82 and 113 DAEs. Among these DEGs, the up-regulated genes in the E-type at 82 DAE included genes encoding beta-glucosidase (bglB) involved in cell wall elongation [46], and genes encoding FERONIA receptor-like kinase (FER) [51] involved in the signal transduction of plant development and the controlling of cell size [56, 57] (S7 Table). And two genes, *bglB* and *FER*, that may be involved in determining the taproot growth rate, were up-regulated. These results suggest that DEGs enriched in the cell wall GO term were responsible for the rapid growth of taproot [13].

We further analyzed the KEGG pathway that may be related to the type-determining of sugar beet cultivar. The plant hormone signal transduction pathway was related to the rapid growth of taproot at 82 DAE. And in the paired comparison of Z-82-vs-E-82, a total number of 107 DEGs were enriched in plant hormone signal transduction pathway with a Q value of 0.05437378. Therefore, plant hormone signal transduction pathway might also be responsible for the different taproot growth rates between the E-type and the Z-type at 82 DAE [59]. The brassinosteroid biosynthesis pathway was enriched in the comparison Z-59-vs-E-59 (S2 Table), suggesting that the different growth strategy between the E-type and the Z-type cultivars was mainly regulated by brassinosteroid. Same as all of other comparisons, plant-pathogen interaction pathway was enriched in the comparison of Z-113-vs-E-113, suggesting that both E-type and Z-type cultivars were subjected to different pathogen pressures at 5 stages. This could be due to the field growth on the farm where pathogens were widespread. In addition, the starch and sucrose metabolism pathway was enriched in the comparisons of Z-134-vs-E-134. Therefore, this pathway could be involved in the different sucrose accumulation between the E-type and the Z-type at 134 DAE. In the comparison of Z-134-vs-E-134, DEGs were involved not only in the starch and sucrose metabolism pathways, but also in the biosynthesis of secondary metabolite and branched-chain amino acid degradation pathways (S2 Table).

A set of plant hormones (i.e. brassinosteroid, auxin, cytokinin and GA) have been analyzed in the taproot development to understand how these hormones regulate the rate of taproot growth in both E-type and Z-type cultivar. At either 59 DAE or 82 DAE, no significant expression difference of *CYP90A1* was observed in both E-type and Z-type. And, the expression level of *TCH4* was higher in the E-type than in the Z-type at 82 DAE (Fig 6B). Since *TCH4* functions as a cell wall-loosening enzyme [75, 76], we postulated that *TCH4* could also be involved in the higher taproot growth rate of two E-type. This postulation is similar to the previous studies in sugar beet [5]. The higher expression of *CYCD3* in the Z-type than in the E-type suggests that cell proliferation in the secondary meristem of the tertiary structure were more active in the Z-type cultivar (Fig 6B). Based on these observations, we infer that the growth strategy of the E-type cultivar was relatively focused on cell enlargement promoted by brassinosteroid signaling, while that of the Z-type was relatively focused on cell division. This conclusion was also supported by the anatomy analysis of taproots at 59 DAE and 82 DAE (data not shown), and the study on taproot sucrose content and cell size of Milford [7]. Therefore, the rapid taproot growth could have been promoted by the different growth strategy in the E-type cultivar.

Both brassinosteroid and auxin signaling work together to regulate the development of taproot. The expression level of 1 *YUCCA* orthologue was higher in the E-type cultivar from 82 to 134 DAE (Fig 7A). But, the expression of *ARF*, *AUX/IAA*, *GH3* and *SUAR* followed an inverse ratio with the rate of taproot growth between both E-type and Z-type cultivars (Fig 7B). Similar results were also observed in the roots of *Arabidopsis* [60]. Therefore the functional integration of auxin and brassinosteroid is important for the determining of the growth rate of taproots.

In taproot development, in addition to brassinosteroid and auxin, cytokinin is another important regulator. We found that the expression level of one of the three *IPT* orthologues was higher in the E-type than in the Z-type cultivar at 59 DAE, while the expression levels of the other two were lower in the E-type than in the Z-type cultivar at 82 DAE (Fig 8A). At 59 DAE, the expression level of 3 *UGT76C1_2* orthologues was lower, while the other 2 orthologues was higher, in the E-type than in the Z-type cultivar. At the growth stages of 82 and 113 DAEs, the expression level of 5 and 3 *UGT76C1_2* orthologues were lower, respectively, in the E-type than in the Z-type cultivar (Fig 8A). These results suggest that, relative to that of the Z-type cultivar, the taproot of the E-type cultivar may possess a higher cytokinin activity at the growth stage of 59 DAE. In addition, the expression level of *B-ARR* was significantly higher in the E-type cultivar at 82 DAE (Fig 8B), suggesting that cytokinin signal transduction pathway also plays a role in regulating the growth rate of taproot.

In taproot development, gibberellin activity was related to the rapid cell enlargement at the temporal scale [6]. During the growth stages from 59 and 134 DAE, the expression levels of 1 *GA*_*20*_*-ox* orthologue and 1 *GA*_*2*_*-ox* orthologue were higher in the E-type than in the Z-type cultivar (Fig 9A). The higher expression level of *GA*_*20*_*-ox* and *GA*_*2*_*-ox* occurred at the same time in the E-type cultivar points to the importance of gibberellin synthesis and homeostasis in taproot development of the E-type cultivar, although more details studies are still needed to dissect the molecular mechanism.

In addition to GO and KEGG enrichment analyses, we also analyzed differentially expressed TF genes. As shown in Table 4, six genes of four TF families, including zf-HD, C2H2, WRKY and Dof, possibly required for rapid taproot growth, were concurrently up-regulated in the comparisons of Z-59-vs-Z-82 and E-59-vs-E-82, when taproot shifted to a rapid growth stage, and in the comparison of Z-82-vs-E-82, when the E-type cultivar taproot grew faster than did the Z-type taproot. Members of *zf-HD* family are related to intercellular trafficking [77], stem growth [78] and hormone signaling [79]. C2H2 family members are the major regulator in cell fate determination in *Arabidopsis* [80], and play an important role in *Medicago truncatula* root enhancement [81]. The members of the WRKY family, GmWRKY13, were involved in promoting lateral root development regulated by the auxin signaling pathway [82]. Furthermore, WRKY22 and WEKY27 were involved in the storage root development of sweet potato. In *Arabidopsis*, WRKY12 plays a role in restraining the ectopic deposition of lignin, xylan and cellulose in secondary walls of pith cells [83]. Members of DOF family, characterized with a conserved region of 50 amino acids with a C2-C2 finger structure involved in the regulation of genes for carbon metabolism [84], undertake an important role in vascular development and interfascicular cambium formation [85–88]. Taken together, these results indicate that transcriptional factors could drive the growth rate of taproot through regulating intercellular trafficking, cell fate determination, auxin signalling, secondary wall strength, and carbohydrate metabolism. Therefore, brassinosteroid, auxin, and cytokinin signaling promoted secondary cambial cell division before taproot turned into rapid growth.

In addition to taproot weight, the difference of sucrose content also existed between two cultivars. The expression level of *Bv2_031430_mgoq*.*t1* (*SPS*) was significantly higher (3.27 folds) at 59 DAE, whereas lower (0.32 fold) at 113 DAEs, in the E-type than in the Z-type cultivar (Fig 9B). The expression level of *Bv7_173910_jyup*.*t1* (*SI*) was higher (2.24 folds) in the E-type than in the Z-type cultivar at 113 DAE (Fig 9B). Therefore, the slower sucrose content increase in the E-type cultivar was correlated to the expression level of *Bv2_031430_mgoq*.*t1* and *Bv7_173910_jyup*.*t1* at 113 DAE, but was not significantly correlated to the expression level of *SPS* and *SI* at 59 DAE.

In order to test the universality of this study, the expression pattern of some crucial genes at 59 and 82 DAEs, in another E-type cultivar ST13092 and another Z-type cultivar ND0905, were compared against the RNA-seq results of the E-type cultivar SD13829 and the Z-type cultivar BS02 (Fig 10B–10D). As shown in Fig 10B–10D, at both growth stages, the expression patterns of these genes in ST13092 and ND0905 were respectively similar to that in SD13829 and BS02. But, the expression of *TCH4_psrc*.*t1* in ND0905, unlike in BS02, was down-regulated at 82 DAE, whereas *TCH4_ydci*.*t1* was significantly up-regulated in ND0905, even though it was not significantly up-regulated in BS02 (Fig 10C). These results imply that different orthologue of *TCH4* are involved in the taproot growth of different cultivars, but *TCH4* always play an important role in both types of cultivars.

GA signaling mainly functions in the transition period before taproot turned into rapid growth. An antagonistic expression pattern of brassinosteroid- and auxin-related genes in taproots was detected in the rapid growth stage of taproots at 82 DAE. In the E-type cultivar SD13829, the growth strategy was relatively focused on cell enlargement promoted by brassinosteroid signaling, whereas in the Z-type cultivar BS02, it was relatively focused on secondarily cambial cell division regulated by cytokinin, auxin and brassinosteroid signaling. The primary cause for high yield and low sucrose content in the two cultivars was not caused by enzymes in sucrose metabolism but the rate of cell enlargement that was promoted by brassinosteroid signaling. In conclusion, our data demonstrate that the weight and sucrose content of taproot in sugar beet rely on its growth strategy, which is controlled by the biosynthesis and signaling of brassinosteroid, auxin, cytokinin and gibberellin.

## Supporting information

S1 Table GO enrichment analyses of DEGs. (XLS)

S2 Table KEGG pathway enrichment analyses of DEGs. (XLS)

S3 Table Analyses of DEGs in plant hormone signal transduction pathway for E-59-vs-E-82. (XLS)

S4 Table Expression level of genes involved plant hormone biosynthesis and metabolism. (XLS)

S5 Table Expression pattern of transcription factor and early response gene of plant hormone signal transduction pathway. (XLS)

S6 Table Expression pattern of sucrose metabolism genes. (XLS)

S7 Table Up-regulated gene expression of GO terms involved in taproot growth. (XLS)

S8 Table GO accession numbers of GO terms. (XLS)

S9 Table Primers used in this study. (XLS)
